# Possible Bat Origin of Severe Acute Respiratory Syndrome Coronavirus 2

**DOI:** 10.3201/eid2607.200092

**Published:** 2020-07

**Authors:** Susanna K.P. Lau, Hayes K.H. Luk, Antonio C.P. Wong, Kenneth S.M. Li, Longchao Zhu, Zirong He, Joshua Fung, Tony T.Y. Chan, Kitty S.C. Fung, Patrick C.Y. Woo

**Affiliations:** The University of Hong Kong, Hong Kong, China (S.K.P. Lau, H.K.H. Luk, A.C.P. Wong, K.S.M. Li, L. Zhu, Z. He, J. Fung, T.T.Y. Chan, P.C.Y. Woo);; United Christian Hospital, Hong Kong (K.S.C. Fung)

**Keywords:** severe acute respiratory syndrome coronavirus 2, SARS-CoV-2, viruses, genomic analysis, recombinant virus, recombination, coronavirus disease, COVID-19, bats, respiratory infections, zoonoses

## Abstract

We showed that severe acute respiratory syndrome coronavirus 2 is probably a novel recombinant virus. Its genome is closest to that of severe acute respiratory syndrome–related coronaviruses from horseshoe bats, and its receptor-binding domain is closest to that of pangolin viruses. Its origin and direct ancestral viruses have not been identified.

Seventeen years after the severe acute respiratory syndrome (SARS) epidemic, an outbreak of pneumonia, now called coronavirus disease (COVID-19), was reported in Wuhan, China. Some of the early case-patients had a history of visiting the Huanan Seafood Wholesale Market, where wildlife mammals are sold, suggesting a zoonotic origin. The causative agent was rapidly isolated from patients and identified to be a coronavirus, now designated as severe acute respiratory syndrome coronavirus 2 (SARS-CoV-2) by the International Committee on Taxonomy of Viruses (*1*). SARS-CoV-2 has spread rapidly to other places; 113,702 cases and 4,012 deaths had been reported in 110 countries/areas as of March 10, 2020 (*2*). In Hong Kong, 130 cases and 3 deaths had been reported.

SARS-CoV-2 is a member of subgenus *Sarbecovirus* (previously lineage b) in the family *Coronaviridae*, genus *Betacoronavirus*, and is closely related to SARS-CoV, which caused the SARS epidemic during 2003, and to SARS-related-CoVs (SARSr-CoVs) in horseshoe bats discovered in Hong Kong and mainland China (*3*–*5*). Whereas SARS-CoV and Middle East respiratory syndrome coronavirus were rapidly traced to their immediate animal sources (civet and dromedaries, respectively), the origin of SARS-CoV remains obscure.

SARS-CoV-2 showed high genome sequence identities (87.6%–87.8%) to SARSr-Rp-BatCoV-ZXC21/ZC45, detected in *Rhinolophus pusillus* bats from Zhoushan, China, during 2015 (*6*). A closer-related strain, SARSr-Ra-BatCoV-RaTG13 (96.1% genome identity with SARS-CoV-2), was recently reported in *Rhinolophus affinis* bats captured in Pu’er, China, during 2013 (*7*). Subsequently, Pangolin-SARSr-CoV/P4L/Guangxi/2017 (85.3% genome identities to SARS-CoV-2) and related viruses were also detected in smuggled pangolins captured in Nanning, China, during 2017 (*8*) and Guangzhou, China, during 2019 (*9*). To elucidate the evolutionary origin and pathway of SARS-CoV-2, we performed an in-depth genomic, phylogenetic, and recombination analysis in relation to SARSr-CoVs from humans, civets, bats, and pangolins (*10*).

## The Study

We downloaded 4 SARS-CoV-2, 16 human/civet-SARSr-CoV, 63 bat-SARSr-CoV and 2 pangolin-SARSr-CoV genomes from GenBank and GISAID (https://www.gisaid.org). We also sequenced the complete genome of SARS-CoV-2 strain HK20 (GenBank accession no. MT186683) from a patient with COVID-19 in Hong Kong. We performed genome, phylogenetic, and recombination analysis as described (*11*).

The 5 SARS-CoV-2 genomes had overall 99.8%–100% nt identities with each other. These genomes showed 96.1% genome identities with SARSr-Ra-BatCoV-RaTG13, 87.8% with SARSr-Rp-BatCoV-ZC45, 87.6% with SARSr-Rp-BatCoV-ZXC21, 85.3% with pangolin-SARSr-CoV/P4L/Guangxi/2017, and 73.8%–78.6% with other SARSr-CoVs, including human/civet-SARSr-CoVs (Table 1).

**Table 1:** Comparison of pairwise percentage amino acid identities between SARS-CoV-2 HK20, 4 other strains of SARS-CoV-2, and other SARSr-CoVs with complete genomes available*

Virus	nsp1	nsp2	nsp3	nsp4	nsp5	nsp6	nsp7	nsp8	nsp9	nsp10	nsp12	nsp13	nsp14	nsp15	nsp16	Spike	NTD	CTD	S2	ORF3	ORF8	E	M	N	Genome
SARS-CoV-2 HKU-SZ-005b_2020/Guangdong/2020	100	100	100	100	100	100	100	100	100	100	100	100	100	100	100	99.3	100	100	100	100	99.2	100	100	100	100
SARS-CoV-2 Wuhan-Hu-1/Hubei/2019	100	100	100	100	100	100	100	100	100	100	100	100	100	100	100	100	100	100	100	100	100	100	100	100	99.9
SARS-CoV-2 WIV02/Hubei/2019	100	100	100	100	100	100	100	100	100	100	100	100	100	100	99.7	99.3	100	100	100	100	100	100	100	100	99.8
SARS-CoV-2 HKU-SZ-002a_2020/Guangdong/2020	100	100	100	100	100	100	100	100	100	100	100	100	100	100	100	99.3	100	100	100	100	99.2	100	100	100	99.8
SARSr-Ra-BatCoV RaTG13/R.affinis/Yunnan/2013	96.7	98.3	96.7	99.6	99.3	99.7	100	100	100	100	99.6	99.7	99.2	97.7	100	96.7	98.6	89.3	99.1	97.8	95.0	100	99.5	99.0	96.1
SARSr-Rp-BatCoV bat-SL-CoVZC45/R.pusillus/Zhejiang/2015	95.6	95.3	93.9	96.8	99.0	97.9	100	97.5	97.3	97.1	95.9	99.2	94.5	89.0	98.0	80.2	66.3	65.5	90.8	90.9	94.2	100	98.6	94.3	87.8
SARSr-Rp-BatCoV bat-SL-CoVZXC21/R.pusillus/Zhejiang/2015	95.6	96.4	93.0	96.2	99.0	97.6	98.8	96.0	96.5	97.8	95.6	98.7	94.7	88.2	98.0	79.6	65.2	64.5	90.5	92.0	94.2	100	98.6	94.3	87.6
Pangolin-SARSr-CoV Guangxi/P4L/2017	90.6	88.4	85.5	90.2	97.1	96.2	100	97.5	97.3	97.1	97.9	98.2	97.0	94.5	97.7	91.7	88.0	86.8	95.6	89.5	87.6	100	98.2	93.8	85.3
SARSr-Rm-BatCoV Longquan-140/R.monoceros/Zhejiang/2012	83.9	82.8	75.9	81.2	95.8	86.6	100	97.5	97.3	94.3	95.7	99.0	94.9	89.0	94.3	75.8	49.3	66.0	90.5	61.5	–	93.4	90.5	89.5	80.1
SARSr-Rs-BatCoV Rs4231/R.sinicus/Yunnan/2013	85.0	67.9	76.3	81.2	96.1	87.2	100	97.0	97.3	97.8	96.4	99.3	95.6	89.0	93.3	76.3	52.2	77.2	87.5	73.8	57.9	93.4	90.1	90.8	79.5
SARSr-Rs-BatCoV Rs3367/R.sinicus/Yunnan/2012	85.0	67.4	76.1	80.4	96.1	86.9	100	97.5	97.3	97.8	96.4	99.3	95.4	88.7	93.0	76.8	53.7	76.6	87.8	74.2	57.9	94.7	90.1	90.5	79.5
SARSr-Rs-BatCoV Rs9401/R.sinicus/Yunnan/2015	84.4	68.3	76.3	80.8	96.1	87.2	100	97.5	97.3	97.8	96.1	99.3	95.6	89.0	93.0	76.6	53.7	76.1	87.7	74.2	57.9	93.4	89.6	90.5	79.5
SARSr-Rs-BatCoV RsSHC014/R.sinicus/Yunnan/2011	85.0	67.9	75.9	81.0	95.8	87.2	100	97.5	97.3	97.8	96.4	99.3	95.1	89.0	92.6	77.0	53.7	77.7	87.9	74.2	58.7	94.7	90.1	90.3	79.4
SARSr-Ra-BatCoV LYRa11/R.affinis/Yunnan/2011	82.2	68.0	76.1	80.8	95.8	86.6	100	97.5	96.5	96.4	96.1	98.8	95.1	89.0	93.0	76.0	49.3	75.1	88.3	76.0	57.9	93.4	89.6	91.0	79.4
SARSr-Rs-BatCoV Rs4237/R.sinicus/Yunnan/2013	83.9	67.7	76.1	81.0	96.1	87.6	98.8	97.5	97.3	97.8	96.7	99.5	95.4	89.0	94.6	75.0	50.0	65.0	89.1	72.0	57.9	94.7	90.5	90.8	79.4
SARSr-Rs-BatCoV Rs4247/R.sinicus/Yunnan/2013	83.9	67.7	76.3	81.0	96.1	87.6	98.8	97.5	97.3	97.8	96.6	99.3	95.6	89.0	94.6	75.2	49.7	65.5	89.3	72.4	57.0	93.4	90.1	90.5	79.4
Human SARS-CoV TOR2/Toronto/Mar2003	84.4	68.3	76.0	80.0	96.1	87.2	98.8	97.5	97.3	97.1	96.4	99.7	95.1	88.7	93.3	75.4	51.9	74.1	87.1	72.4	–	94.7	90.5	90.5	79.4
Human SARS-CoV GZ02/Guangdong/Feb2003	83.9	68.3	76.1	79.8	96.1	87.2	98.8	97.5	97.3	97.1	96.4	99.3	95.1	88.7	93.6	75.7	52.6	74.6	87.3	73.1	29.7	94.7	90.5	90.5	79.4
SARSr-Rs-BatCoV Rs4081/R.sinicus/Yunnan/2012	85.0	67.9	76.3	81.0	96.1	87.6	100	97.5	97.3	97.8	96.4	99.2	95.8	89.0	94.3	74.4	48.1	64.0	89.0	72.0	58.7	94.7	90.1	90.3	79.3
SARS-As-BatCoV As6526/Aselliscus stoliczkanus/Yunnan/2014	84.4	67.9	76.3	81.0	95.8	87.2	100	97.5	97.3	97.8	96.1	99.3	95.6	89.0	94.6	75.1	49.7	66.0	88.9	71.6	58.7	94.7	91.0	90.0	79.3
Human SARS-CoV BJ01/Beijing	84.4	68.3	76.0	80.4	96.1	87.2	98.8	97.5	97.3	97.1	96.4	99.5	95.1	88.7	93.3	75.6	52.2	74.1	87.3	72.4	–	94.7	90.5	90.5	79.3
Civet SARS-CoV SZ3/Shenzhen/2013	84.4	68.5	76.1	80.6	96.1	87.2	98.8	97.5	97.3	97.1	96.4	99.5	95.1	88.7	93.6	75.7	51.9	75.1	87.3	72.4	29.7	94.7	90.1	90.5	79.3
Civet SARS-CoV SZ16/Shenzhen/2013	84.4	68.5	76.0	80.6	96.1	87.2	98.8	97.5	97.3	97.1	96.4	99.5	95.1	88.7	93.6	75.7	51.9	75.1	87.3	72.4	29.7	94.7	90.1	90.5	79.3
SARSr-Ra-BatCoV YN2018A/R.affinis/Yunnan?/2016	84.4	67.9	76.3	80.8	96.1	86.9	100	97.5	97.3	97.8	96.5	99.5	95.4	89.0	64.1	75.2	49.7	65.5	89.1	71.3	57.9	94.7	89.6	90.3	79.2
SARSr-Rs-BatCoV Rs4255/R.sinicus/Yunnan/2013	85.0	67.9	76.1	80.8	96.1	87.2	100	97.5	97.3	97.8	96.1	99.3	95.6	88.7	94.3	74.5	48.1	65.0	89.0	72.0	58.7	94.7	89.2	90.3	79.2
SARSr-Rs-BatCoV Rp3/R.sinicus/Guangxi/2004	82.8	67.7	75.4	80.6	95.8	87.2	100	96.0	96.5	97.8	96.2	99.2	95.4	88.4	93.3	75.0	49.3	65.5	89.3	74.5	56.2	94.7	90.1	90.3	79.2
SARSr-Rs-BatCoV Rs4084/R.sinicus/Yunnan/2012	84.4	68.3	76.3	80.4	95.8	87.2	100	97.5	97.3	97.8	96.2	98.5	95.1	88.7	92.6	76.9	53.7	77.2	87.9	74.2	–	94.7	90.1	90.0	79.2
Civet SARS-CoV PC4–227/Guangdong/2004	84.4	68.2	76.0	80.6	96.1	86.9	98.8	97.0	96.5	97.1	96.2	99.3	94.9	88.7	93.0	75.4	52.2	75.1	86.7	72.4	–	94.7	90.1	90.0	79.2
SARSr-Rs-BatCoV HuB2013/R.sinicus/Hubei/2013	83.9	69.3	76.0	81.0	96.1	86.6	100	97.5	97.3	97.8	96.1	99.0	95.6	88.2	94.0	74.9	47.8	66.0	89.4	75.3	49.6	93.4	91.0	91.0	79.1
SARSr-Rm-BatCoV Rm1/R.macrotis/Hubei/2004	80.0	69.1	74.8	80.6	95.8	86.6	100	97.0	96.5	97.1	95.9	98.8	94.9	87.0	93.0	74.4	47.6	65.0	89.0	74.5	57.0	93.4	91.0	90.0	79.1
SARSr-Rs-BatCoV Anlong-103/Guizhou/R.sinicus/2013	84.4	68.2	75.8	80.8	96.1	86.9	98.8	97.5	97.3	97.1	96.5	99.3	95.6	89.0	93.6	75.1	50.2	64.5	89.1	72.0	29.7	94.7	89.6	89.6	79.1
Civet SARS-CoV civet020/Guangdong/2004	84.4	68.0	75.9	80.6	96.1	87.2	97.6	97.0	97.3	97.1	96.4	99.3	94.7	88.7	93.3	75.4	52.2	75.1	86.7	72.4	29.7	94.7	90.1	90.0	79.1
SARSr-Rs-BatCoV HKU3-12/R.sinicus/Hong Kong/2007	83.9	68.8	75.3	81.8	95.8	86.6	100	97.5	96.5	97.8	95.9	98.7	94.7	88.2	93.3	75.8	49.0	66.5	90.5	73.5	57.0	94.7	91.0	89.1	79.0
SARSr-Rs-BatCoV HKU3-7/R.sinicus/Guangdong/2006	83.9	68.5	75.5	81.4	95.8	87.2	100	96.5	97.3	97.8	95.8	99.2	94.5	87.9	94.0	75.7	49.0	66.5	90.3	73.5	57.0	93.4	90.1	90.3	79.0
SARSr-Rs-BatCoV HKU3–8/R.sinicus/Guangdong/2006	83.9	68.5	75.6	81.4	95.4	87.2	100	96.5	97.3	97.8	95.8	99.2	94.5	87.9	94.0	75.9	49.3	66.5	90.5	73.5	–	92.1	89.6	90.3	79.0
SARSr-Rs-BatCoV HKU3–1/R.sinicus/Hong Kong/2005	83.9	69.0	75.2	81.4	95.4	87.2	100	96.5	97.3	97.8	95.6	98.7	94.7	88.2	93.3	75.7	49.0	66.5	90.3	73.1	57.0	94.7	91.0	89.5	79.0
SARSr-Rs-BatCoV HKU3–3/R.sinicus/Hong Kong/2005	83.9	69.0	75.2	81.4	95.4	87.2	100	96.5	97.3	97.8	95.6	98.7	94.7	88.2	93.3	75.7	49.0	66.5	90.3	73.1	57.0	94.7	91.0	89.5	79.0
SARSr-Rs-BatCoV HKU3–9/R.sinicus/Hong Kong/2006	83.9	69.0	75.2	81.4	95.1	87.2	100	96.5	97.3	97.8	95.6	98.8	94.7	87.6	93.3	75.7	49.0	66.5	90.2	72.7	57.0	94.7	91.0	89.3	79.0
SARSr-Rf-BatCoV Rf4092/R.ferrumequinum/Yunnan/2012	84.4	67.7	76.1	81.0	96.1	87.2	98.8	96.5	97.3	97.8	96.5	99.5	95.1	89.0	93.0	72.6	39.9	66.0	88.6	71.3	28.9	94.7	89.6	90.5	79.0
SARSr-Rs-BatCoV Anlong-112/Guizhou/R.sinicus/2013	83.9	68.2	76.0	80.8	96.1	87.2	98.8	97.5	97.3	97.8	96.5	99.3	95.6	89.0	93.6	75.1	50.2	64.5	89.1	72.0	29.7	94.7	89.6	89.6	79.0
SARSr-Rs-BatCoV HKU3–2/R.sinicus/Hong Kong/2005	83.9	69.0	75.3	81.4	95.4	87.2	100	96.5	97.3	97.8	95.6	98.8	94.7	87.6	93.3	75.7	49.0	66.5	90.3	73.1	57.0	94.7	91.0	89.8	78.9
SARSr-Rs-BatCoV HKU3–13/R.sinicus/Hong Kong/2007	83.9	69.0	75.1	81.4	95.4	87.2	100	96.5	97.3	97.8	95.6	98.8	94.5	87.6	93.3	75.7	49.0	66.5	90.2	72.7	57.0	94.7	91.0	89.3	78.9
UNVERIFIED: SARSr-Rp-BatCoV F46/R.pusillus/Yunnan/2012	81.7	68.3	75.8	80.8	96.1	86.2	98.8	97.5	96.5	97.1	96.4	99.3	95.6	89.6	93.6	72.6	39.9	66.0	88.9	70.9	26.6	94.7	89.6	90.5	78.9
Human SARS-CoV GZ0401/Guangdong/Dec2003	84.4	68.2	45.9	80.6	96.1	87.2	97.6	97.0	97.3	97.1	96.2	99.3	94.9	88.7	93.3	75.3	51.9	75.1	86.7	64.4	29.7	94.7	84.7	90.0	78.9
SARSr-Rl-BatCoV SC2018/Rhinolophus sp./2016	83.3	69.3	74.9	81.2	95.8	86.9	100	97.5	97.3	97.8	96.1	98.8	95.4	86.7	92.3	74.4	47.4	66.0	88.9	70.9	55.4	94.7	90.1	90.7	78.8
SARSr-Rf-BatCoV YNLF_31C/R.ferrumequinum/Yunnan/2013	78.9	69.0	75.8	80.8	95.8	86.6	97.6	97.0	97.3	97.1	96.2	99.2	95.6	88.7	93.0	73.4	49.3	65.5	86.2	70.9	29.7	94.7	89.6	90.0	78.7
Civet SARS-CoV B039/Guangdong/2004	84.4	68.2	75.9	80.6	96.1	87.2	98.8	97.0	97.3	97.1	96.4	99.5	94.7	88.7	93.3	75.4	52.2	75.1	86.7	72.4	29.7	94.7	90.1	90.0	78.6
Civet SARS-CoV civet007/Guangdong/2004	84.4	68.0	75.9	80.6	96.1	87.2	98.8	97.0	97.3	97.1	96.4	99.5	94.9	88.7	93.0	75.3	51.9	75.1	86.6	72.0	29.7	94.7	90.1	90.0	78.6
Civet SARS-CoV A022/Guangdong/2004	84.4	68.2	76.0	80.6	96.1	87.2	98.8	97.0	97.3	97.1	96.4	99.5	94.9	88.7	92.6	75.3	51.9	75.1	86.6	72.0	29.7	94.7	90.1	89.8	78.5
SARSr-Rf-BatCoV Rf1/R.ferrumequinum/Hubei/2004	78.9	68.8	75.3	81.6	96.1	86.6	100	97.0	97.3	97.1	96.2	99.0	94.9	88.2	91.9	73.6	50.0	66.0	86.2	68.7	31.3	93.4	89.6	89.1	78.4
SARSr-Rp-BatCoV Rp/Shaanxi2011/R.pusillus/Shaanxi/2011	84.4	69.3	75.8	81.2	96.1	86.6	100	97.5	97.3	97.8	96.1	99.2	96.0	88.4	93.6	74.7	48.0	66.0	89.1	72.4	58.7	93.4	90.5	91.0	78.4
SARSr-Rf-BatCoV Jiyuan-84/R.ferrumequinum/ Henan/2012	77.8	68.8	75.3	81.4	95.4	86.9	100	97.0	97.3	97.1	96.2	99.0	95.8	89.0	91.9	73.3	49.7	66.0	85.8	70.2	28.9	93.4	89.6	89.8	78.3
SARSr-Cp-BatCoV Cp/Yunnan2011/C. plicata/Yunnan/2011	81.7	68.0	76.5	81.0	96.1	86.6	100	97.0	96.5	97.8	96.0	98.0	94.7	89.3	93.0	74.5	49.0	65.5	88.7	72.0	57.9	94.7	91.4	90.3	78.2
SARSr-Ra-BatCoV YN2018B/R.affinis/Yunnan?/2016	85.0	68.2	76.3	80.6	96.1	87.2	100	97.5	97.3	97.8	96.4	99.3	95.6	89.0	93.0	76.6	53.4	76.1	87.8	73.8	57.9	94.7	90.1	90.8	78.1
SARSr-Rs-BatCoV Rs7327/R.sinicus/Yunnan/2014	84.4	67.9	76.2	80.8	96.1	87.2	100	97.5	97.3	97.8	96.2	99.2	95.6	89.0	93.0	76.7	53.7	76.1	87.8	73.8	57.0	94.7	90.1	90.5	78.1
SARSr-Rs-BatCoV Rs4874/R.sinicus/Yunnan/2013	85.0	67.9	76.3	81.0	96.1	87.6	100	97.5	97.3	97.8	96.2	98.7	95.3	88.7	93.3	76.5	52.2	76.6	87.9	74.2	57.9	94.7	89.6	90.3	78.1
SARSr-Rs-BatCoV WIV16/R.sinicus/Yunnan/2013	85.0	67.9	76.2	81.0	96.1	87.6	100	97.5	97.3	97.8	96.1	99.3	94.7	88.7	93.3	76.5	52.2	76.6	87.9	74.2	57.9	94.7	90.1	90.3	78.1
SARSr-Ra-BatCoV YN2018D/R.affinis/Yunnan?/2016	85.0	68.2	76.3	81.0	96.1	87.2	100	97.5	97.3	97.8	96.5	99.3	95.4	89.0	94.0	74.4	48.1	64.0	89.1	72.7	57.9	94.7	76.1	90.5	77.9
SARSr-Rf-BatCoV SX2013/R.ferrumequinum/Shanxi/2013	78.3	69.1	75.3	81.2	95.8	86.6	100	96.5	97.3	97.1	95.9	99.0	95.8	89.0	91.9	73.3	49.7	66.0	85.8	70.9	30.5	92.1	89.6	89.8	77.9
SARSr-Rf-BatCoV HeB2013/R.ferrumequinum/Hebei/2013	78.9	69.1	75.1	81.6	95.8	86.6	100	97.0	97.3	97.1	96.1	99.0	95.8	88.7	91.9	73.4	49.7	66.0	85.9	70.5	30.5	93.4	89.6	89.8	77.7
SARSr-Rs-BatCoV YN2013/R.sinicus/Yunnan/2013	83.9	67.9	76.1	80.6	96.1	87.2	100	97.5	97.3	97.8	96.4	99.3	95.8	88.4	93.3	75.1	50.9	64.0	89.0	71.3	29.7	94.7	91.0	90.8	77.5
SARSr- Rs-BatCoV Rs672/2006/R.sinicus/Guizhou/2006	85.0	67.9	68.3	80.4	95.4	87.2	100	97.5	97.3	97.8	96.5	98.8	95.6	88.2	94.3	74.2	47.8	64.0	89.0	71.6	58.7	94.7	89.6	90.5	77.5
SARSr-Rs-BatCoV GX2013/R.sinicus/Guangxi/2013	83.3	68.3	75.9	80.2	95.8	87.2	100	97.5	96.5	97.1	96.5	99.3	95.4	89.0	94.0	75.1	49.7	66.5	89.0	72.7	29.7	94.7	91.4	89.6	77.3
SARSr-Rf-BatCoV 16BO133/R.ferrumequinum/Korea/2016	78.9	69.1	67.6	81.2	95.1	85.9	100	96.5	97.3	97.1	96.1	98.5	94.9	88.2	92.6	71.2	39.0	65.0	86.6	68.0	–	93.4	89.6	88.8	76.9
SARSr-Rf-BatCoV JL2012/R.ferrumequinum/Jilin/2012	78.9	69.0	74.8	81.2	94.8	85.9	98.8	96.5	97.3	97.1	96.2	98.3	95.1	87.9	92.6	71.2	39.5	65.0	86.6	68.0	–	92.1	89.6	89.3	76.9
SARSr-Rf-BatCoV JTMC15/R.ferrumequinum/Jilin/2013	78.9	69.0	67.2	81.2	94.8	85.9	98.8	96.5	97.3	97.1	96.2	98.3	94.9	88.2	92.6	71.2	39.3	65.0	86.6	68.0	–	90.8	90.1	89.3	76.4
SARSr-Rs-BatCoV BtKY72/Rhinolophus sp./Kenya/2007	82.2	63.2	71.6	82.2	95.4	82.1	94.0	95.5	97.3	95.0	95.0	96.7	92.8	87.3	90.6	71.6	46.6	72.7	82.5	63.6	19.1	94.7	86.9	86.2	75.5
SARSr-Rb-BatCoV BM48–31/BGR/2008/R.blasii/Bulgaria/2008	81.7	62.4	71.3	81.0	94.1	83.8	95.2	96.5	98.2	95.0	95.2	97.7	93.5	89.9	88.6	71.3	44.1	69.7	83.4	62.5	–	92.1	85.9	87.6	75.2

*SARS-CoV-2 strains are highlighted in tan, and SARSr-CoV strains that had the highest end identity for each gene with SARS-CoV-2 are highlighted in red. SARSr-CoV strains were listed in descending order according to complete genome identities to SARS-CoV-2 HK20/Hong Kong/2020.. Pairwise amino acid identities could not be calculated for some strains because of completely or partially truncated ORF8. CTD, C-terminal domain; E, envelope; M, matrix; N, nucleocapsid; nsp, nonstructural protein; NTD, N-terminal domain; ORF, open reading frame; S, spike; SARS-CoV- 2, severe acute respiratory syndrome coronavirus 2; SARSr-CoV, severe acute respiratory syndrome–related coronavirus; –, not applicable..

Most predicted proteins of SARS-CoV-2 showed high amino acid sequence identities with that of SARSr-Ra-BatCoV RaTG13, except the receptor-binding domain (RBD) region. SARS-CoV-2 possessed an intact open reading frame 8 without the 29-nt deletion found in most human SARS-CoVs. The concatenated conserved replicase domains for coronavirus species demarcation by the International Committee on Taxonomy of Viruses showed >92.9% aa identities (threshold >90% for same species) between SARS-CoV-2 and other SARSr-CoVs, supporting their classification under the same coronavirus species (Table 2) (*1*).

**Table 2:** Percentage amino acid identity between 7 conserved domains of the replicase polyprotein for species demarcation in SARS-CoV-2 and selected members of the subgenus Sarbecovirus*

Virus	% Amino acid identity compared with that for SARS-CoV-2							
	ADRP	nsp5	nsp12	nsp13	nsp14	nsp15	nsp16	Seven concatenated domains
Human SARS-CoV TOR2/Toronto/Mar2003	79.3	96.1	96.4	99.8	95.1	88.7	93.3	95.0
Civet SARS-CoV SZ3/Shenzhen/2013	79.3	96.1	96.4	99.7	95.1	88.7	93.6	95.0
Civet SARS-CoV PC4–136/Guangdong/2004	78.4	96.1	96.4	99.3	94.7	88.7	93.3	94.8
Human SARS-CoV GZ0402	78.4	95.8	96.2	99.5	94.9	51.7	93.3	94.8
SARSr-Rs-BatCoV HKU3–1/R.sinicus/Hong Kong/2005	79.3	95.4	95.6	98.8	94.7	88.2	93.3	94.5
SARSr-Rp-BatCoV Rp/Shaanxi2011/R.pusillus/Shaanxi/2011	78.4	96.1	96.1	99.3	96.0	88.4	93.6	95.0
SARSr-Rs-BatCoV Rs672/2006/R.sinicus/Guizhou/2006	80.2	95.4	96.5	99.0	95.6	88.2	94.3	95.0
SARSr-Rs-BatCoV WIV1	80.2	95.8	96.2	99.5	95.4	89.0	93.0	95.0
SARSr-Rf-BatCoV YNLF_31C/R.ferrumequinum/Yunnan/2013	80.2	95.8	96.2	99.3	95.6	88.7	93.0	95.0
SARSr-Rf-BatCoV 16BO133/R.ferrumequinum/Korea/2016	79.3	95.1	96.1	98.7	94.9	88.2	92.6	94.6
SARSr-Rf-BatCoV JTMC15/R.ferrumequinum/Jilin/2013	79.3	94.8	96.2	98.5	94.9	88.2	92.6	94.5
SARSr-Rs-BatCoV BtKY72/Rhinolophus sp./Kenya/2007	74.8	95.4	95.0	96.8	92.8	87.3	90.6	92.9
SARSr-Rm-BatCoV Longquan-140/R.monoceros/Zhejiang/2012	79.3	95.8	95.7	99.2	94.9	89.0	94.3	94.8
SARSr-Rb-BatCoV BM48–31/BGR/2008/R.blasii/Bulgaria/2008	78.4	94.1	95.2	97.8	93.5	89.9	88.6	93.5
SARSr-Rp-BatCoV ZXC21/R.pusillus/Zhejiang/2015	88.3	99.0	95.6	98.8	94.7	88.2	98.0	95.4
SARSr-Rp-BatCoV ZC45/R.pusillus/Zhejiang/2017	92.8	99.0	95.9	99.3	94.5	89.0	98.0	95.8
Pangolin-SARSr-CoV Guangxi/P4L/2017	85.5	97.1	97.9	98.2	97.0	94.5	97.7	96.9
SARSr-Ra-BatCoV RaTG13/R.affinis/Yunnan/2013	96.7	99.3	99.6	99.7	99.2	97.7	100	99.2

*ADRP, ADP-ribose-1′′ phosphatase; nsp, nonstuctural protein; SARS-CoV-2, severe acute respiratory syndrome coronavirus 2; SARSr-CoV, severe acute respiratory syndrome–related coronavirus.

Unlike other members of the subgenus *Sarbecovirus*, SARS-CoV-2 has a spike protein that contains a unique insertion that results in a potential cleavage site at the S1/S2 junction, which might enable proteolytic processing that enhances cell–cell fusion. SARS-CoV-2 was demonstrated to use the same receptor, human angiotensin-converting enzyme 2 (hACE2), as does SARS-CoV (*7*). The predicted RBD region of SARS-CoV-2 spike protein, corresponding to aa residues 318–513 of SARS-CoV (*12*), showed the highest (97% aa) identities with pangolin-SARSr-CoV/MP789/Guangdong and 74.1%–77.7% identities with human/civet/bat-SARSr-CoVs known to use hACE2 (Table 1). Moreover, similar to the human/civet/bat-SARSr-CoV hACE2-using viruses, the 2 deletions (5 aa and 12 aa) found in all other SARSr-BatCoVs (*10*) were absent in SARS-CoV-2 RBD (Appendix Figure 1). Of the 5 critical residues needed for RBD-hACE2 interaction in SARSr-CoVs (*13*), 3 (F472, N487, and Y491) were present in SARS-CoV-2 RBD and pangolin SARSr-CoV/MP789/2019-RBD.

**Figure 1:**
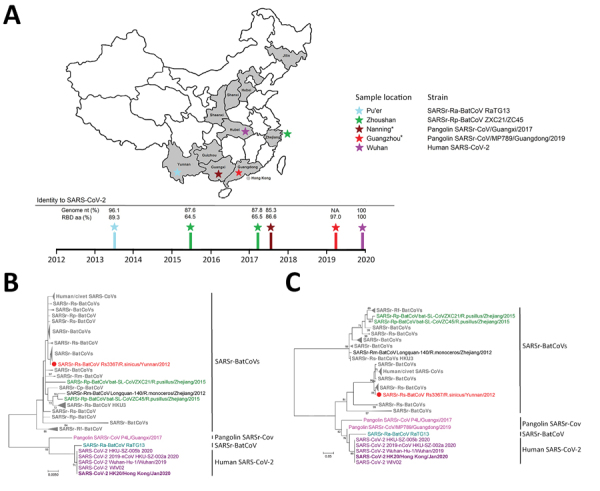
Geographic and phylogenetic comparisons of SARS-CoV-2 isolates with closely related viruses. A) Locations in China where SARS-CoV-2 first emerged (Wuhan), and where closely related viruses were found, including SARSr-Ra-BatCoV RaTG13 (Pu’er), Pangolin-SARSr-CoVs (Guangzhou and Nanning), and SARSr-Rp-BatCoV ZC45 (Zhoushan). Time of sampling and percentage genome identities to SARS-CoV-2 are shown. *Guangzhou and Nanning. The geographic origin of smuggled pangolins remains unknown. B, C) Phylogenetic analyses of RdRp (B) and RBD (C) domains of SARSr-CoVs. Trees were constructed by using maximum-likelihood methods with Jones-Taylor-Thornton plus gamma plus invariant sites (RdRp) and Whelan and Goldman plus gamma (RBD) substitution models. A total of 745 aa residues for RdRp and 177 aa residues for RBD were included in the analyses. Numbers at nodes represent bootstrap values, which were calculated from 1,000 trees. Only bootstrap values >70% are shown. Purple indicates SARS-CoV-2 (strain HK20 in bold); teal indicates SARSr-Ra-BatCoV RaTG13; pink indicates pangolin SARSr-CoVs; green indicates SARSr-Rp-BatCoVs ZXC21 and ZC45; red indicates SARSr-Rs-BatCoV Rs3367; black indicates SARSr-Rs-BatCoV Longquan-140; gray indicates remaining SARSr-BatCoVs. Dots indicate SARSr-BatCoVs reported to use angiotensin-converting enzyme 2 as receptor. Scale bars indicate estimated number of amino acid substitutions per 200 aa residues for RdRp and per 20 aa residues for RBD. SARS-CoV-2, severe acute respiratory syndrome coronavirus 2; SARSr-CoV, severe acute respiratory syndrome–related coronavirus; NA, not available; RBD, receptor-binding domain; RdRp, RNA-dependent RNA polymerase.

Phylogenetic analysis showed that the RNA-dependent RNA polymerase gene of SARS-CoV-2 is most closely related to that of SARSr-Ra-BatCoV RaTG13, whereas its predicted RBD is closest to that of pangolin-SARSr-CoVs (Figure 1). This finding suggests a distinct evolutionary origin for SARS-CoV-2 RBD, possibly as a result of recombination. Moreover, the SARS-CoV-2 RBD was also closely related to SARSr-Ra-BatCoV RaTG13 and the hACE2-using cluster containing human/civet-SARSr-CoVs and Yunnan SARSr-BatCoVs previously successfully cultured in VeroE6 cells (*4*,*5*).

**Figure 2:**
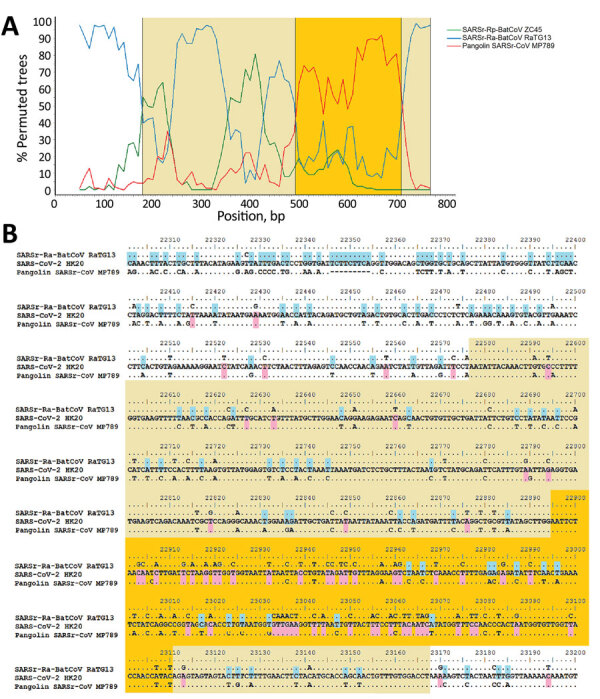
Bootscan analysis and nucleotide sequence alignment for SARS-CoV-2 isolates and closely related viruses. A) Boot scan analysis using the partial spike gene (positions 22397–23167) of SARS-CoV-2 strain HK20 as query sequence. Bootscanning was conducted with Simplot version 3.5.1 (https://sray.med.som) (F84 model; window size, 100 bp; step, 10 bp) on nucleotide alignment, generated with ClustalX (http://www.clustal.org). B) Multiple alignment of nucleotide sequences from genome positions 22300 to 23700. Yellow indicates receptor binding domain; orange indicates receptor binding motif; pink indicates bases conserved between SARS-CoV-2 HK20 and Pangolin-SARSr-CoV/MP789/Guangdong/2019; and blue indicates bases conserved between SARS-CoV-2 HK20 and SARSr-Ra-BatCoVs RaTG13.

To identify putative recombination events, we performed sliding window analysis using SARS-CoV-2-HK20 as query and SARSr-Ra-BatCoV RaTG13, pangolin-SARSr-CoV/P4L/Guangxi/2017, SARSr-Rp-BatCoV ZC45, SARSr-Rs-BatCoV Rs3367, and SARSr-Rs-BatCoV Longquan-140 as potential parents (Figure 2; Appendix Figures 2). A similarity plot showed that SARS-CoV-2 is most closely related to SARSr-Ra-BatCoV RaTG13 in the entire genome, except for its RBD, which is closest to pangolin-SARSr-CoV/MP789/Guangdong, and shows potential recombination breakpoints. Moreover, different regions of SARS-CoV-2 genome showed different similarities to pangolin-SARSr-CoV/P4L/Guangxi/2017, SARSr-Rp-BatCoV ZC45, SARSr-Rs-BatCoV Rs3367, and SARSr-Rs-BatCoV Longquan-140, as supported by phylogenetic analysis (Appendix Figures 2, 3).

Sequence alignment around the RBD supported potential recombination between SARSr-Ra-BatCoV RaTG13 and pangolin-SARSr-CoV/MP789/Guangdong/2019 and the receptor-binding motif region showing exceptionally high sequence similarity to that of pangolin-SARSr-CoV/MP789/Guangdong/2019. This finding suggested that SARS-CoV-2 might be a recombinant virus between viruses closely related to SARSr-Ra-BatCoV RaTG13 and pangolin-SARSr-CoV/MP789/Guangdong/2019.

## Conclusions

Despite the close relatedness of SARS-CoV-2 to bat and pangolin viruses, none of the existing SARSr-CoVs represents its immediate ancestor. Most of the genome region of SARS-CoV-2 is closest to SARSr-Ra-BatCoV-RaTG13 from an intermediate horseshoe bat in Yunnan, whereas its RBD is closest to that of pangolin-SARSr-CoV/MP789/Guangdong/2019 from smuggled pangolins in Guangzhou. Potential recombination sites were identified around the RBD region, suggesting that SARS-CoV-2 might be a recombinant virus, with its genome backbone evolved from Yunnan bat virus–like SARSr-CoVs and its RBD region acquired from pangolin virus–like SARSr-CoVs.

Because bats are the major reservoir of SARSr-CoVs and the pangolins harboring SARSr-CoVs were captured from the smuggling center, it is possible that pangolin SARSr-CoVs originated from bat viruses as a result of animal mixing, and there might be an unidentified bat virus containing an RBD nearly identical to that of SARS-CoV-2 and pangolin SARSr-CoV. Similar to SARS-CoV, SARS-CoV-2 is most likely a recombinant virus originated from bats.

The ability of SARS-CoV-2 to emerge and infect humans is likely explained by its hACE2-using RBD region, which is genetically similar to that of culturable Yunnan SARSr-BatCoVs and human/civet-SARSr-CoVs. Most SARSr-BatCoVs have not been successfully cultured in vitro*,* except for some Yunnan strains that had human/civet SARS-like RBDs and were shown to use hACE2 (*4*,*5*). For example, SARSr-Rp-BatCoV ZC45, which has an RBD that is more divergent from that of human/civet-SARSr-CoVs, did not propagate in VeroE6 cells (*6*). Factors that determine hACE2 use among SARSr-CoVs remain to be elucidated.

Although the Wuhan market was initially suspected to be the epicenter of the epidemic, the immediate source remains elusive. The close relatedness among SARS-CoV-2 strains suggested that the Wuhan outbreak probably originated from a point source with subsequent human-to-human transmission, in contrast to the polyphyletic origin of Middle East respiratory syndrome coronavirus (*14*). If the Wuhan market was the source, a possibility is that bats carrying the parental SARSr-BatCoVs were mixed in the market, enabling virus recombination. However, no animal samples from the market were reported to be positive. Moreover, the first identified case-patient and other early case-patients had not visited the market (*15*), suggesting the possibility of an alternative source.

Because the RBD is considered a hot spot for construction of recombinant CoVs for receptor and viral replication studies, the evolutionarily distinct SARS-CoV-2 RBD and the unique insertion of S1/S2 cleavage site among *Sarbecovirus* species have raised the suspicion of an artificial recombinant virus. However, there is currently no evidence showing that SARS-CoV-2 is an artificial recombinant, which theoretically might not carry signature sequences. Further surveillance studies in bats are needed to identify the possible source and evolutionary path of SARS-CoV-2.

Appendix Additional information on possible bat origin of severe acute respiratory syndrome coronavirus virus 2.
