# Supplementary material for: Possible Bat Origin of Severe Acute Respiratory Syndrome Coronavirus 2
Source: Emerg Infect Dis. 2020 Jul;26(7):1542–7. doi: 10.3201/eid2607.200092 (PMC7323513; doi:10.3201/eid2607.200092)
Supplement: Appendix — Additional information on possible bat origin of severe acute respiratory syndrome coronavirus virus 2. [file 20-0092-Techapp-s1.pdf]

Article DOI: https://doi.org/10.3201/eid2607.200092

Possible Bat Origin of Severe Acute Respiratory Syndrome Coronavirus 2

Appendix

Appendix Table. Severe acute respiratory syndrome coronavirus strains used in this study*

Virus	GenBank accession no.
SARSr-Rp-BatCoV ZC45/R.pusillus/Zhejiang/2015	MG772934.1
SARSr-Rp-BatCoV ZXC21/R.pusillus/Zhejiang/2015	MG772933.1
SARSr-Rm-BatCoV Longquan-140/R.monoceros/Zhejiang/2012	KF294457.1
SARSr-Rs-BatCoV HKU3-1/R.sinicus/Hong Kong/2005	DQ022305.2
SARSr-Rs-BatCoV HKU3-12/R.sinicus/Hong Kong/2007	GQ153547.1
SARSr-Rs-BatCoV HKU3-11/R.sinicus/Hong Kong/2007	GQ153546.1
SARSr-Rs-BatCoV HKU3-9/R.sinicus/Hong Kong/2006	GQ153544.1
SARSr-Rs-BatCoV HKU3-6/R.sinicus/Hong Kong/2005	GQ153541.1
SARSr-Rs-BatCoV HKU3-5/R.sinicus/Hong Kong/2005	GQ153540.1
SARSr-Rs-BatCoV HKU3-4/R.sinicus/Hong Kong/2005	GQ153539.1
SARSr-Rs-BatCoV HKU3-3/R.sinicus/Hong Kong/2005	DQ084200.1
SARSr-Rs-BatCoV HKU3-2/R.sinicus/Hong Kong/2005	DQ084199.1
SARSr-Rs-BatCoV HKU3-13/R.sinicus/Hong Kong/2007	GQ153548.1
SARSr-Rs-BatCoV HKU3-10/R.sinicus/Hong Kong/2006	GQ153545.1
SARSr-Rs-BatCoV HKU3-8/R.sinicus/Guangdong/2006	GQ153543.1
SARSr-Rs-BatCoV HKU3-7/R.sinicus/Guangdong/2006	GQ153542.1
SARSr-Rs-BatCoV Rp3/R.sinicus/Guangxi/2004	DQ071615.1
SARSr-Rs-BatCoV Rs4247/R.sinicus/Yunnan/2013	KY417148.1
SARSr-Rs-BatCoV Rs4237/R.sinicus/Yunnan/2013	KY417147.1
SARSr-Rs-BatCoV Rs4081/R.sinicus/Yunnan/2012	KY417143.1
SARSr-Rs-BatCoV Rs4255/R.sinicus/Yunnan/2013	KY417149.1
SARS-As-BatCoV As6526/Aselliscus stoliczkanus/Yunnan/2014	KY417142.1
SARSr-Rs-BatCoV HuB2013/R.sinicus/Hubei/2013	KJ473814.1
SARSr-Rf-BatCoV JTMC15/R.ferrumequinum/Jilin/2013	KU182964.1
SARSr-Rm-BatCoV 279/2005/R.macrotis/Hubei/2005	DQ648857.1
SARSr-Ra-BatCoV YN2018D/R.affinis/Yunnan?/2016	MK211378.1
SARSr-Ra-BatCoV YN2018C/R.affinis/Yunnan?/2016	MK211377.1
SARSr-Rm-BatCoV Rm1/R.macrotis/Hubei/2004	DQ412043.1
SARSr-Rs-BatCoV Rs3367/R.sinicus/Yunnan/2012	KC881006.1
SARSr-Rs-BatCoV RsSHC014/R.sinicus/Yunnan/2011	KC881005.1
SARSr-Rs-BatCoV WIV1	KF367457.1
SARSr-Rs-BatCoV Rs9401/R.sinicus/Yunnan/2015	KY417152.1
SARSr-Rs-BatCoV Rs4874/R.sinicus/Yunnan/2013	KY417150.1
SARSr-Ra-BatCoV YN2018A/R.affinis/Yunnan?/2016	MK211375.1
SARSr-Rs-BatCoV Anlong-103/Guizhou/R.sinicus/2013	KY770858.1
SARSr-Rs-BatCoV Anlong-112/Guizhou/R.sinicus/2013	KY770859.1
Human SARS-CoV TOR2/Toronto/Mar2003	NC_004718
Human SARS-CoV GZ02/Guangdong/Feb2003	AY390556.1
SARSr-Rs-BatCoV Rs7327/R.sinicus/Yunnan/2014	KY417151.1
SARSr-Rs-BatCoV Rs4231/R.sinicus/Yunnan/2013	KY417146.1
SARSr-Rf-BatCoV Rf4092/R.ferrumequinum/Yunnan/2012	KY417145.1
UNVERIFIED: SARSr-Rp-BatCoV F46/R.pusillus/Yunnan/2012	KU973692.1
SARSr-Rs-BatCoV WIV16/R.sinicus/Yunnan/2013	KT444582.1
SARSr-Ra-BatCoV YN2018B/R.affinis/Yunnan?/2016	MK211376.1
Human SARS-CoV BJ01/Beijing	AY278488.2
Civet SARS-CoV SZ16/Shenzhen/2013	AY304488.1
Civet SARS-CoV SZ3 /Shenzhen/2013	AY304486.1
Civet SARS-CoV GD69/Guangdong/May2003	AY313906.1
SARSr-Rf-BatCoV YNLF_34C/R.ferrumequinum/Yunnan/2013	KP886809.1
SARSr-Rf-BatCoV YNLF_31C/R.ferrumequinum/Yunnan/2013	KP886808.1
SARSr-Rs-BatCoV Rs4084/R.sinicus/Yunnan/2012	KY417144.1
Civet SARS-CoV PC4-227/Guangdong/2004	AY613950.1
Civet SARS-CoV PC4-136/Guangdong/2004	AY613949.1
Civet SARS-CoV PC4-13/Guangdong/2004	AY613948.1
Human SARS-CoV GZ0402	AY613947.1

Virus	GenBank accession no.
SARSr-Rf-BatCoV Rf1/R.ferrumequinum/Hubei/2004	DQ412042.1
SARSr-Rf-BatCoV 273/2005/R.ferrumequinum/Hubei/2005	DQ648856.1
SARSr-Ra-BatCoV LYRa11/R.affinis/Yunnan/2011	KF569996.1
SARSr-Rl-BatCoV Rl-SC2018/Rhinolophus sp./2016	MK211374.1
Civet SARS-CoV A001	FJ959407.1
Civet SARS-CoV civet020/Guangdong/2004	AY572038.1
SARSr-Rp-BatCoV Rp/Shaanxi2011/R.pusillus/Shaanxi/2011	JX993987.1
SARSr-Rf-BatCoV Jiyuan-84/R.ferrumequinum/ Henan/2012	KY770860.1
Human SARS-CoV GZ0401/Guangdong/Dec2003	AY568539.1
Civet SARS-CoV civet007/Guangdong/2004	AY572034.1
Civet SARS-CoV civet010/Guangdong/2004	AY572035.1
Civet SARS-CoV B039/Guangdong/2004	AY686864.1
SARSr-Cp-BatCoV Cp/Yunnan2011/Chaerephon plicata/Yunnan/2011	JX993988.1
Civet SARS-CoV A022/Guangdong/2004	AY686863.1
SARSr-Rf-BatCoV SX2013/R.ferrumequinum/Shanxi/2013	KJ473813.1
SARSr-Rf-BatCoV HeB2013/R.ferrumequinum/Hebei/2013	KJ473812.1
SARSr- Rs-BatCoV Rs672/2006/R.sinicus/Guizhou/2006	FJ588686.1
SARSr-Rs-BatCoV YN2013/R.sinicus/Yunnan/2013	KJ473816.1
SARSr-Rs-BatCoV GX2013/R.sinicus/Guangxi/2013	KJ473815.1
SARSr-Rf-BatCoV JL2012/R.ferrumequinum/Jilin/2012	KJ473811.1
SARSr-Rf-BatCoV 16BO133/R.ferrumequinum/Korea/2016	KY938558.1
SARSr-Rs-BatCoV BtKY72/Rhinolophus sp./Kenya/2007	KY352407.1
SARSr-Rb-BatCoV BM48-31/BGR/2008/R.blasii/Bulgaria/2008	GU190215.1
SARSr-Ra-BatCoV RaTG13/Yunnan/2013	MN996532.1
SARS-CoV-2 Wuhan-Hu-1/Hubei/2019	MN908947.1
SARS-CoV-2 WIV02/Hubei/2019	MN996527.1
SARS-CoV-2 HKU-SZ_002a/Guangdong/2020	MN938384.1
SARS-CoV-2 HKU-SZ_005b/Guangdong/2020	MN975262.1
Pangolin SARSr-CoV Guangxi/P4L/2017	MT040333.1
Pangolin SARSr-CoV MP789	MT084071.1
SARS-CoV-2 HK20/Hong Kong/2020	MT186683

*SARS-CoV-2, severe acute respiratory syndrome coronavirus 2; SARSr-CoV, severe acute respiratory syndrome–related coronavirus.

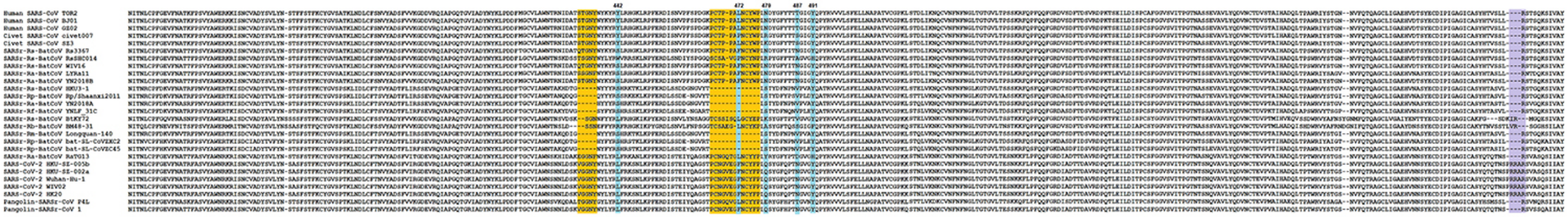

Appendix Figure 1. Multiple alignment of amino acid sequences of the receptor-binding domain of the spike proteins of SARS-CoV-2; human, pangolin, and civet SARSr-CoVs; and corresponding sequences of SARSr-BatCoVs in different *Rhinolophus* species. Asterisks indicate positions that have fully conserved residues. Dashes indicate deletions. Amino acid deletions in some SARSr-BatCoVs are highlighted in orange; the 5 critical residues for receptor binding in human SARS-CoV at positions 442, 472, 479, 487, and 491 are highlighted in blue, and the polybasic cleavage site is highlighted in purple. SARS-CoV-2, severe acute respiratory syndrome coronavirus 2; SARSr-CoV, severe acute respiratory syndrome–related coronavirus.

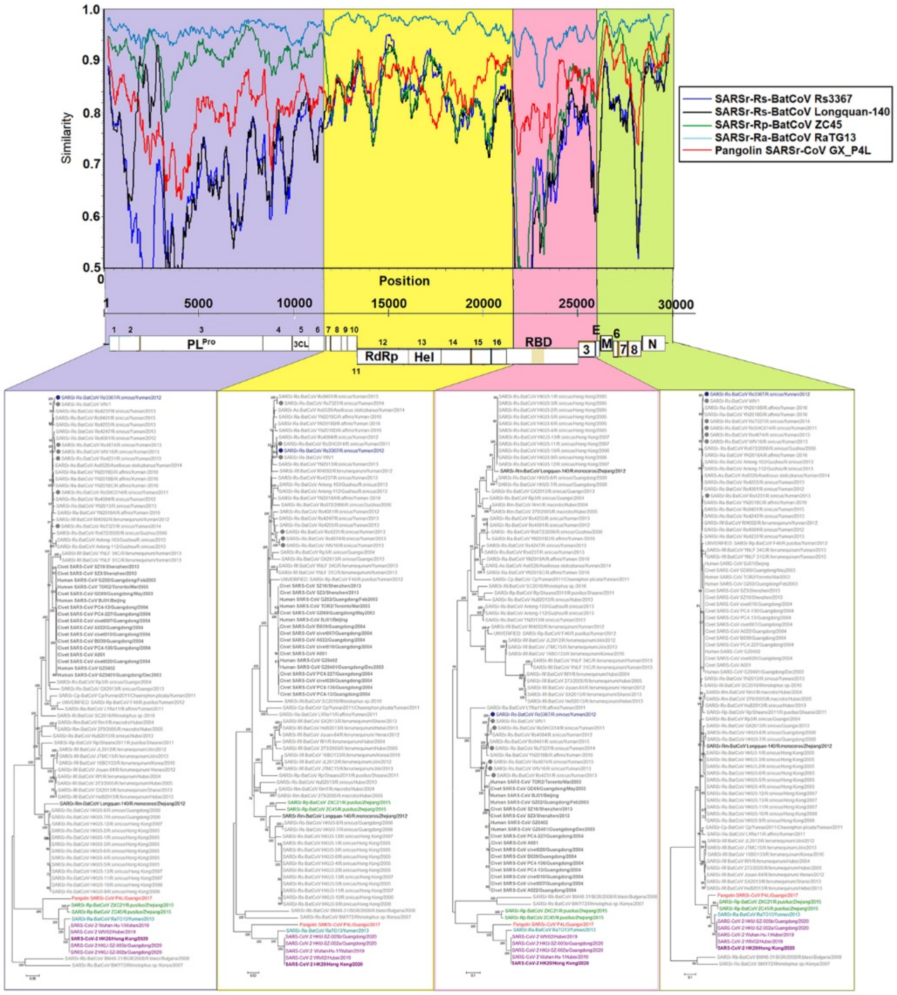

Appendix Figure 2. Simplot analysis using the genome sequence of SARS-CoV-2 strain HK20 as the query sequence. Simplot analysis was conducted with Simplot version 3.5.1 (F84 model; window size, 400 bp; step, 40 bp) on nucleotide alignment, generated with ClustalX. The teal line denotes SARSr-Ra-BatCoV RaTG13, the red line denotes pangolin-SARSr-CoV-GX_P4L, the green line denotes SARSr-Rp-BatCoV strain ZC45, the blue line denotes SARSr-Rs-BatCoV strain Rs3367, and the black line denotes SARSr-Rm-BatCoV strain Longquan-140. Phylogenetic trees were constructed by maximum-likelihood method using the generalized time reversible + gamma + invariant substitution model based on nucleotides sequences for the regions from the 5'end to position 11502, position 11502 to 21509, position 21509 to 25928 and position 25928 to 3' end. Bootstrap values were calculated from 1,000 trees. Only bootstrap values of >70% are shown. E, envelope; Hel 1, helicase; M, matrix; N, nucleocapsid; RBD, receptor-binding protein; PL; papain-like protease; RdRp, RNA-dependent RNA polymerase; SARS-CoV-2, severe acute respiratory syndrome coronavirus 2; SARSr, severe acute respiratory syndrome–related coronavirus.

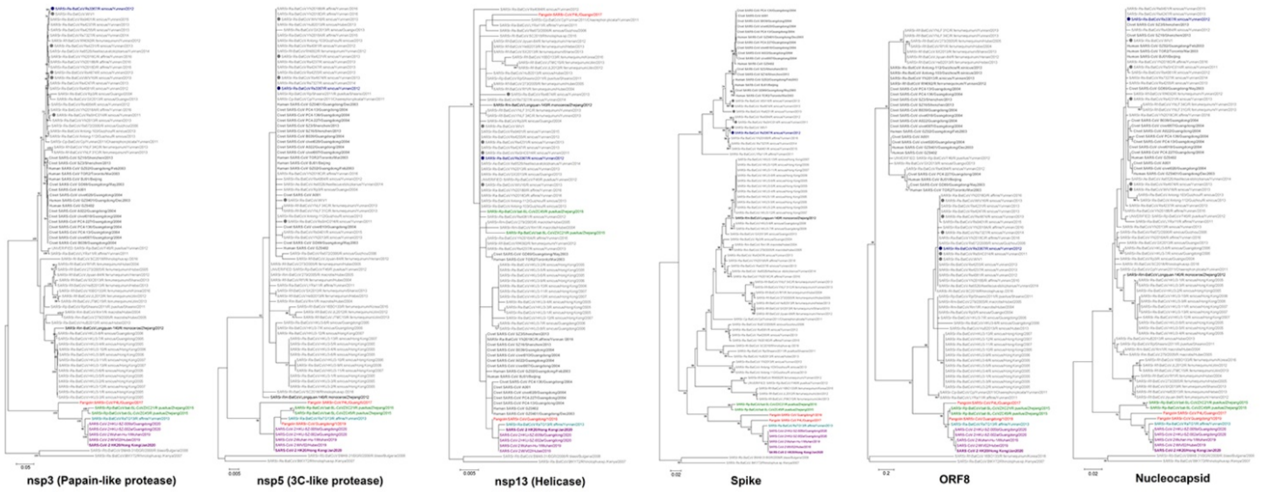

Appendix Figure 3. Phylogenetic analysis of nonstructural protein 3 (nsp3), nsp5, nsp13, spike protein, open reading frame 8 (ORF8), and nucleocapsid protein of severe acute respiratory syndrome–related coronavirus causing the SARS epidemic during 2003. Viruses were isolated from bats, pangolins, civets, and humans. Trees were constructed by using the maximum-likelihood method and Jones-Taylor-Thornton + gamma (nsp3, nsp5, nsp13, and nucleocapsid); leaving group + gamma + invariant (spike); and Jones-Taylor-Thornton + invariant (ORF8) substitution models. Bootstrap values were calculated from 1,000 trees. Amino acid positions 1666, 306, 600, 1225, 108 and 414 in nsp3, nsp5, nsp13, spike, ORF8, and nucleocapsid, respectively, were included in the analysis. Scale bar indicates estimated number of amino acid substitutions per 20, 200, 200, 50, 5, and 50 positions in nsp3, nsp5, nsp13, spike, ORF8, and nucleocapsid, respectively.
